# HeLP-Diabetes: randomised controlled trial protocol

**DOI:** 10.1186/s12913-015-1246-9

**Published:** 2015-12-29

**Authors:** Elizabeth Murray, Charlotte Dack, Maria Barnard, Andrew Farmer, Jinshuo Li, Susan Michie, Kingshuk Pal, Steve Parrott, Jamie Ross, Michael Sweeting, Bindie Wood, Lucy Yardley

**Affiliations:** eHealth Unit, Research Department of Primary Care and Population Health, University College London, Upper Floor 3, Royal Free Hospital, Rowland Hill Street, London, NW3 2PF UK; Psychology Department, University of Bath, Claverton Down, Bath, BA2 7AY UK; The Whittington Hospital NHS Trust, Magdala Avenue, London, N19 5NF UK; Department of Primary Care Health Sciences, University of Oxford, New Radcliffe House, Radcliffe Observatory Quarter, Woodstock Road, Oxford, OX2 6GG UK; Department of Health Sciences, Seebohm Rowntree Building, University of York, Heslington, York, YO10 5DD UK; UCL Centre for Behaviour Change, University College London, 1-19 Torrington Place, London, WC1E 7HB UK; Department of Public Health and Primary Care, University of Cambridge, Strangeways Research Laboratory, Wort’s Causeway, Cambridge, CB1 8RN UK; Centre for Application of Health Psychology, University of Southampton, Building 44, Highfield Campus, Southampton, SO17 1BJ UK

**Keywords:** Diabetes mellitus, Type 2, Self-care, Internet, Randomized controlled trial, Primary health care, Family practice

## Abstract

**Background:**

Type 2 Diabetes Mellitus (T2DM) is common, affecting nearly 400 million people worldwide. Achieving good health for people with T2DM requires active self-management; however, uptake of self-management education is poor, and there is an urgent need to find better, more acceptable, cost-effective methods of providing self-management support. Web-based self-management support has many potential benefits for patients and health services. The aim of this trial is to determine the effectiveness and cost-effectiveness of a web-based self-management support programme for people with T2DM.

**Methods:**

This will be a multi-centre individually randomised controlled trial in primary care, recruiting adults with T2DM who are registered with participating general practices in England. Participants will be randomised to receive either an evidence-based, theoretically informed, web-based self-management programme for people with T2DM which addresses medical, emotional, and role management, called Healthy Living for People with type 2 Diabetes (HeLP-Diabetes) or a simple information website. The joint primary outcomes are glycated haemoglobin (HbA1c) and diabetes-related distress, measured by the Problem Areas In Diabetes (PAID) questionnaire. Secondary outcomes include cardiovascular risk factors, depression and anxiety, and self-efficacy for self-management of diabetes. Health economic data include health service use, costs due to the intervention, and EQ-5D for calculation of Quality Adjusted Life Years (QALYS). Data will be collected at baseline, 3 months and 12 months, with the primary endpoint at 12 months. Practice nurses, blinded to patient allocation, collect clinical data; patients complete online questionnaires for patient reported measures. A sample size of 350 recruited participants allows for attrition of up to 15 % and will provide 90 % power of detecting at a 5 % significance level a true average difference in the PAID score of 4.0 and 0.25 % change in HbA1c (both small effect sizes). The analysis will follow a pre-specified analysis plan, based on comparing the groups as randomised (intention-to-treat).

**Discussion:**

The findings of this trial are likely to be of interest to policy makers, clinicians, and commissioners, all of whom are actively seeking additional forms of self-management support for people with T2DM.

**Trial registration:**

The Trial Registration number is ISRCTN 02123133; date of registration 14.2.13.

## Background

Type 2 diabetes (T2DM) is one of the commonest long term conditions affecting nearly 400 million people worldwide [1] and over 3 million in the UK [2]. T2DM can cause severe complications including cardiovascular disease, blindness, renal failure and neuropathy, and can reduce life expectancy by 8 – 10 years [3]. In the UK, approximately 12 % of deaths of people between the ages of 20 – 79 years are attributable to diabetes and about 10 % of the NHS budget (£9 billion per annum) is spent on diabetes [4]. Many of these costs are due to preventable complications. The key to good clinical outcomes in people with diabetes is self-management which can reduce both the incidence and impact of complications. Structured education is known to promote self-management and reduce the incidence of diabetes complications [5–7]. In 2008, the National Institute for Health and Care Excellence (NICE) advised that all patients with diabetes should be offered structured education at the time of diagnosis with annual reinforcement [3]. NICE advised that such structured education or self-management programmes (SMP) should improve outcomes through addressing health beliefs, optimising metabolic control, addressing cardiovascular risk factors, facilitating behaviour change, improving quality of life and reducing depression as well as enhancing the relationship between the patient and their healthcare professionals [3].

Examples of existing self-management programmes for people with type 2 diabetes in the UK include DESMOND [7], X-PERT [8] and Co-Creating Health [9]. Although these programmes have shown initial benefits, there are concerns that benefits may not be sustained in the long term [10]. There are additional concerns that group-based programmes such as these may not suit all patients who need self-management training. People who work, have caring responsibilities at home, have mobility problems, or who find group interactions difficult may all have difficulty attending. Thus there is an urgent need to find cost-effective and acceptable methods of delivering sustainable self-management education for people with type 2 diabetes in the UK. One possibility is the use of web-based or computer-based self-management programmes. These have many potential advantages, including convenience, accessibility and anonymity. For people with home access to the internet (84 % of UK households in 2014) [11], such programmes can be accessed at any time of day or night so can be fitted in around responsibilities at home or work. Information can be presented accessibly using simple graphics or audio-visual techniques and can be easily updated as new research becomes available. Programmes can be highly interactive, responding to data entered by individual users to provide a tailored, personalised experience. They can offer structured behaviour change support including self-assessment, goal-setting, monitoring and feedback. Users can gain emotional support from reading about others’ experiences with similar problems (personal stories), participating in online forums, or using online support tools such as computerised cognitive behavioural therapy or mindfulness training. Unlike face-to-face interventions, computerised programmes can be permanently available and provide both on-going support as well as meeting changing needs as the disease progresses. The marginal costs per additional user are low, so such interventions have the potential to be highly cost-effective. A recent Cochrane systematic review of such programmes suggested they can improve some health outcomes including glycaemic control, weight and lipids, but there were insufficient data to draw any conclusions about their impact on emotional well-being, quality of life or cost-effectiveness. None of these programmes addressed all the areas specified by NICE for self-management education, and none were developed in the UK [12].

We have developed a web-based self-management programme for people with type 2 diabetes known as HeLP-Diabetes, or Healthy Living for People with type 2 diabetes. HeLP-Diabetes was developed as part of a National Institute for Health Research (NIHR) Programme Grant for Applied Research. The content and development of HeLP-Diabetes was based on five main principles: first, we adopted the Corbin and Strauss model of the work required for self-management of a long-term condition, which includes medical management (e.g. adopting healthy behaviours, working with health professionals, managing medicines), emotional management (e.g. managing the strong negative emotions resulting from being diagnosed with a long term condition including anger, guilt, shame and despair), and role management (e.g. managing the disruption of one’s biographical narrative) [13]. This model gave us an overarching framework of patients’ requirements. Secondly we undertook extensive qualitative work with our target users, defined as patients with type 2 diabetes and health professionals who care for these patients, to identify user needs and wants from such a programme. Effective self-management requires a partnership between patients and health professionals so it was important to ensure that the programme was acceptable to both groups of users. User input continued throughout the development process with user panels providing iterative comments on materials as they were developed and refined (participatory design), ensuring that the final programme was highly acceptable to both patients and health professionals. Thirdly, we reviewed the behaviour change literature to identify behaviour change techniques that were most likely to be effective in helping patients achieve sustainable behaviour change. Fourthly, we applied the principles of Normalization Process Theory (NPT) to maximise the likelihood of the programme being easily implemented and integrated into routine NHS care [14]. Finally, we ensured that all information provided in the programme was evidence-based and compatible with current NICE guidelines.

## Methods/design

### Aims and objectives

The aims of the trial are to:

1. Determine the effect of HeLP-Diabetes on clinical outcomes and health related quality of life in people with T2DM
2. Determine the incremental cost-effectiveness of the intervention from the perspectives of health and personal social services and wider public sector resources.

Hypothesis: that use of the intervention will improve diabetes-related quality of life and health status.

### Design

Multi-centre, two-arm individually randomised controlled trial in primary care.

### Ethics and trial registration

This trial received ethical approval from Camden and Islington Research Ethics Committee reference 12/LO/1571. The Trial Registration number is ISRCTN 02123133.

### Setting

General practices in England.

### Participants

Inclusion criteria:

- Adults aged 18 or over, with type 2 diabetes.

Exclusion criteria:

- Unable to provide informed consent e.g. due to psychosis, dementia or severe learning difficulties
- Terminally ill with less than 12 months life expectancy
- Unable to use a computer due to severe mental or physical impairment
- Insufficient mastery of spoken or written English to use the intervention

Participants do not have to have home internet access or prior experience of using the internet to participate. Participants with previous or current experience of self-management education are eligible to participate.

### Recruitment

Approximately 20 practices will be recruited through 5 Primary Care Research Networks (PCRN) in England (South West Central, East of England, South East, Greater London) and the North Central London Research Consortium (NoCLOR). Practices will be assessed for their feasibility (e.g. large enough diabetes register to be able to invite a minimum of 300 eligible participants; staff are trained in Good Clinical Practice (GCP); 2 members of staff available to carry out the study so that 1 can support the participant in use of the intervention and the other can remain blinded for data collection) prior to agreement to act as a participating site. Once a practice has agreed to participate and completed site set-up procedures they will commence patient recruitment.

Patient recruitment will follow standard opt-in procedures. Each practice will have a register of patients with type 2 diabetes as they need this for the Quality and Outcomes Framework. A nurse or other qualified health professional will review the electronic medical record of each of the patients on this register with a view to screening out ineligible patients. All remaining potentially eligible patients will be sent a letter from their GP, inviting them to participate in the study. A participant information sheet, consent form, expression of interest and stamped addressed envelope will be included. Patients who are interested in participating will be asked to return the expression of interest form to the trial manager.

In addition posters advertising the study will be put up in each practice so that patients can ask a clinician directly for more information about taking part.

On receipt of the expression of interest form the nurse will contact the patient and offer them an appointment at the practice. At this appointment, patients will be given an opportunity to discuss the pros and cons of participation, and those that agree to participate will be asked to sign the written consent form. After signing the consent form, participants will be asked to complete baseline data collection procedures prior to randomisation. Randomisation marks the point of entry into the trial.

### Randomisation

Randomisation will be performed only after all baseline data collection has been completed. Randomisation will be at the level of the individual participant. It will be stratified by recruitment centre and will be performed centrally using a web-based randomisation system provided by an independent contractor (Sealed Envelope http://www.sealedenvelope.com/). This system will send an email to the trial manager when a patient has been randomised detailing which arm the patient has been allocated to. This will then be forwarded to the nurse who will be introducing the control and intervention websites to the patients in visit 2.

### Intervention

The intervention consists of facilitated and supported access to HeLP-Diabetes. There are three components to the supported access: first an introductory session, secondly supportive follow-up phone calls, and thirdly, on-going discussion of patient’s self-management goals in routine appointments for diabetes-related matters.

In the introductory session practice nurses will give the patient a booklet containing the uniform resource locator (URL) for the programme, the participant’s log in details, and information about the content of the website and how best to use it. Nurses will show the patient how to access the website, and introduce them to the main content areas. The nurse will discuss with the patient what the patient’s most pressing needs are and use this to guide the patient toward certain sections (for example, improving diet, being more physically active, or managing emotions). Follow-up phone calls will be offered to support the patient in use of the programme. Nurses and doctors in participating practices will be encouraged to refer to the programme in consultations with participating patients and to integrate information from the programme into management plans.

HeLP-Diabetes is a web-based programme with multiple components. There are information sections  including how diabetes is treated, possible complications of diabetes, possible impacts of diabetes on relationships at home and at work, dealing with unusual situations like parties, holidays, travelling or shift work, and what lifestyle modifications will improve health. There are sections addressing skills and behaviour change, including behaviour change modules on eating healthily, losing weight, being more physically active, smoking cessation, moderating alcohol consumption, managing medicines, glycaemic control and blood pressure control. These all include motivating information on the benefits of behaviour change, self-assessment quizzes for patients to assess whether and how much they need to change, and opportunities for goal-setting and self-monitoring. The third strand of components focuses on emotional well-being and contains self-help tools based on cognitive behavioural therapy and mindfulness. There are multiple personal stories (used with license from healthtalk.org), and a moderated forum for users to interact with each other.

### Comparator

From an NHS perspective the important research question is whether the proposed intervention can improve health outcomes when compared to current practice. However, to improve acceptability to participants and help maintain blinding, all participants will have access to a website. Participants in the control arm will have access to a simple information website, based on the information available on the Diabetes UK and NHS Choices websites. These participants will also be given a booklet with the URL and user log in details, and will also have the introductory session with the nurse explaining how to use the website and offer of follow-up phone calls. However, as the website will have no behaviour change components, no emotional support and only simple information, it is unlikely that there will be many opportunities to refer to it in diabetes-related appointments.

### Outcomes and outcome measures

#### Primary outcomes

The outcomes reflect our aims of improving clinical outcomes and health related quality of life. We have selected two joint primary outcomes: glycated haemoglobin (HbA1c) and diabetes-related distress measured by the Problem Areas in Diabetes (PAID) scale [15]. PAID has 20 items focusing on areas that cause difficulty for people living with diabetes, including social situations, food, friends and family, diabetes treatment, relationships with health care professionals and social support. It has been the subject of a number of reviews comparing available quality of life measures for diabetes. Eigenmann assessed available measures against criteria of: reliability; content, face, construct, criterion and convergent validity; responsiveness to change; interpretability; response burden; acceptability; and availability and concluded that PAID was one of three measures that met all criteria [16]. It is sensitive to change and has been widely used to evaluate self-management programmes for people with T2DM including the influential DESMOND trial [7].

#### Secondary outcomes

Secondary outcomes have been selected to reflect the proposed pathway of action of our intervention and allow health economic analysis and can be categorised as clinical, patient-reported, or economic.

#### Clinical outcomes include:

- Systolic and diastolic blood pressure
- Body mass index;
- Total cholesterol and HDL (not fasting)
- Completion of “9 essential processes” [3] (= weight, BP, smoking status, measurement of serum creatinine, cholesterol and HbA1c, urinary albumin and assessment of eyes and feet).

Patient-reported outcomes:

- Depression and anxiety measured using the Hospital Anxiety and Depression Scale (HADS) [17]
- Diabetes-related self-efficacy measured using the Diabetes Management Self-Efficacy Scale (DMSES) [18]
- Satisfaction with treatment measured using the Diabetes Satisfaction with Treatment Questionnaire status and change version (DTSQs & DTSQc) [19].

Economic outcomes:

- Cost of developing the intervention
- Cost of supported access
- Costs of training NHS staff both in using the intervention and training patients to use the intervention
- Costs of maintaining and updating the intervention
- Health care and social service utilisation during the study period
- EQ-5D to calculate QALYs [20]
- Clinical parameters required for modelling long term cost-effectiveness of the intervention (detailed below).

In addition we will use automated software to record each participant’s use of the intervention (number and frequency of log-ins, pages visited).

### Data collection

Data to describe our patient population will be collected at baseline and will include demographic and clinical data.

Demographic data will include: age, gender, highest educational attainment, ethnicity, current employment status, presence or absence of home internet access, level of expertise in computer use, and current or previous participation in diabetes self-management education.

Baseline clinical data to be obtained from the medical record will include:

- date of diagnosis of diabetes
- HbA1c, blood pressure, total cholesterol, HDL cholesterol and smoking status at time of diagnosis
- presence or absence and date of diagnosis of complications of diabetes including ischaemic heart disease, myocardial infarction, congestive cardiac failure, atrial fibrillation, peripheral vascular disease, amputation, cerebro-vascular disease, retinopathy, renal failure and neuropathy
- a list of current medications
- Additional clinical data on height (cm) weight (kg), systolic and diastolic blood pressure, current smoking status, and current levels of HbA1c, total cholesterol and HDL cholesterol will also be obtained during the baseline visit

Baseline patient reported outcomes include:

- PAID
- HADS
- DTSQ
- EQ-5D
- DMSES

Baseline health economic data includes:

- Clinical data as above
- Health service utilisation in the 12 months prior to baseline visit.

Follow up data will be collected at 3 and 12 months with 12 months as the primary outcome point. At follow-up data will be collected on the clinical and patient reported outcomes listed above, along with attendance at alternative self-management education. Demographic data will only be collected at baseline.

Data on use of health service utilisation will be collected for the past 12 months at baseline, the past 3 months at 3 month follow-up and the past 9 months at 12 month follow-up. Data on completion of the “9 essential processes” will be collected from the GP record for the 12 months prior to randomisation and the 12 months after randomisation at the 12 month follow-up point to avoid triggering behaviour change amongst the study nurses.

Standard operating procedures (SOPs) will cover every aspect of data collection and nurses will be trained in these procedures. Adherence to SOPs will be monitored. Participants will complete self-reported questionnaires (demographics, PAID, HADS, DMSES, DTSQ, EQ-5D and community and social service use) online, prior to the nurse recording clinical outcomes and taking blood for HbA1c and lipids. Clinical data will be entered directly into the online database by the nurse. Data on utilisation of primary and secondary health care services will be extracted from the clinical record by the nurse.

### Concealment of allocation, blinding and protection against bias

Baseline data will be obtained prior to randomisation. Randomisation will be performed centrally and allocation will not be revealed to the participants who will have been informed that the trial is comparing two forms of web-based education for diabetes (a simple/straightforward version vs. a more detailed/complicated version). Practice nurses will be provided with similar looking booklets for both comparator and intervention websites. There are potential problems with contamination (e.g. two members of the same household being randomised to different interventions with individual randomisation but cluster randomisation has greater potential to introduce bias by affecting GP behaviour (which patients they refer to the trial) and patient behaviour (participation). We will monitor the extent to which contamination occurs by determining the proportion of participants who had contact with another trial participant with a different treatment allocation.

The risk of bias in collection of follow-up data will be minimised by using standardised data collection instruments with participants completing self-assessment questionnaires before seeing the nurse to record clinical data. Clinical data will be collected by research nurses who will be trained to adhere to detailed SOPs developed in collaboration with PRIMENT CTU. Follow-up data will be collected by different nurses to those who undertake the baseline visits and training. Nurses collecting follow-up data will be blind to allocation. Blood pressure will be recorded using automated electronic sphygmanometers.

### Adherence and loss to follow-up

Fidelity of the intervention will be promoted through training practice nurses and provision of detailed SOPs to guide how nurses introduce participants to the intervention. Use of the intervention and comparator websites will be recorded automatically.

Every effort will be made to promote follow-up which will be co-ordinated by the trial manager centrally. At each follow-up point (3 and 12 months) participants will be sent up to two automated emails containing an embedded hotlink to the follow-up questionnaires. Non-responders will be sent an additional (third) personalised email from the trial manager, explaining how important follow up data are for the study, and the link will also be resent. After this, participants are sent a letter through the post, containing both the link and pencil-and-paper versions of the questionnaires with a stamped addressed envelope for returning it, in case participants prefer to complete the questionnaires offline. Finally, the trial manager will contact the patient by phone to explore reasons for non-response, encourage response, and if necessary, ask the patient to complete the PAID verbally over the phone.

Practice nurses are reminded of the need to offer participants follow-up appointments and to complete the Case Report Form (CRF) for clinical and health economic data.

### Sample size

We hypothesise that use of the intervention will improve both PAID scores and HbA1c. The analyses will gain power through adjustment for baseline levels. We have back-calculated the relevant effective standard deviations (SDs) from a previous trial as 0.676 for HbA1c and 10.75 for PAID, substantially lower than the SDs of cross-sectional measures of around 1.4 and 16 respectively because of the correlation between baseline and subsequent measures. We intend to recruit 350 participants; with attrition of up to 15 % we anticipate at least 300 patients for the primary analysis. This will give us 90 % power of detecting at a 5 % significance level a true average difference in the PAID score of 4.0 and 0.25 % change in HbA1c. These are both small effect sizes. Since HbA1c and PAID are joint primary outcomes measuring very different aspects of T2DM both will be tested at a 5 % significance level. Results from both outcomes will be presented side-by-side in any publication irrespective of their statistical significance. This follows similar practice in other trials in this area [21].

### Analysis

The analysis will follow a pre-specified analysis plan based on comparing the groups as randomised (intention-to-treat). Follow-up HbA1c will be adjusted for initial levels and other baseline covariates including age, gender, centre, participation in other self-management programmes, pre-existing cardiovascular disease and duration of diabetes. PAID and other outcome measures will be analysed similarly. Sub-group analysis will be undertaken by baseline glycaemic control and duration of diabetes, treating all potential effect modifiers as continuous. For the purpose of presentation, results will be shown for those with good vs. poor baseline glycaemic control (HbA1c 7.5 % or greater vs. 7.4 % or less). Missing follow-up data will be multiply imputed where possible using other outcome data (e.g. 3 m data when 12 m data are missing) and other sensitivity analyses investigating the potential for bias undertaken.

The amount of use the intervention receives (e.g. number of log-ins) will be further investigated as a potential mediator for efficacy. Using instrumental variable methods with randomisation as the instrument, an unbiased estimate of the average causal effect of the intervention on the primary outcomes (HbA1c and PAID) will be obtained in a subgroup who would use the intervention a certain amount [22].

If contamination (where members of the control group have access to the active intervention e.g. through a family member in the intervention group) does occur it will be dealt with in the analysis by:

- Our main (primary) analysis will be a full intention-to-treat analysis on the whole trial population
- We will report the extent of any contamination
- We will undertake a sensitivity analysis using non-contaminated controls in the control arm only. However as this loses the benefits of randomisation we will also
- Undertake a Complier Average Causal Effect (CACE) analysis which respects randomisation. For this CACE analysis we would label contaminated control participants as potential non-compliers [23].

### Economic analysis

#### Incremental cost-effectiveness

The determination of within-trial incremental cost-effectiveness of facilitated access to HeLP-Diabetes compared to usual care for patients with T2DM will follow NICE guidance by adopting both a health and personal social services and a wider public sector resource perspective [24, 25]. The components of the analysis are health outcomes, costs of the active and control intervention, and the potential impact on diabetes care and complications. QALYs will be calculated using area under the curve analysis from the baseline, 3- and 12- month follow-ups, adjusting for baseline levels. Intervention costs will be measured directly. NHS and social care costs will be calculated from the self-reported usage data collected from patients and the data extracted from the electronic medical record by nurses.

#### Costs of the intervention

Costs of the intervention to the NHS are made up of two major components: development of the intervention and facilitation/implementation costs. The resources required for the development of the intervention will be monitored during the development process. Once the intervention has been developed it will need ongoing maintenance and updating; these costs will be recorded. The economic evaluation will include the maintenance and hosting costs, with research costs separated from treatment costs. Development costs will be considered sunk costs and not included in the analysis as these would not be incurred if the intervention were rolled out in practice.

#### Facilitated access and implementation costs

Implementation costs will be largely made up of staff time health professional time, and patient time. A proforma will be constructed so that time spent by the implementation staff in the project can be attributed to the different stages of the implementation process. The individual help given to each patient and health professional will be collected and coded to each practice, and costed on the basis of the full economic costs of the staff involved. Estimates of the additional time required from the general practice staff will be obtained. The costs of health professional input from the practices will be based on national average rates using PSSRU (Personal Social Services Research Unit) estimates. Costs calculated for the different implementation and facilitated access activities will be estimated for each participating practice and compared to patient activity. The data will be used to construct models of the potential implementation costs and population benefits for a “typical” Clinical Commissioning Group and how these costs and effects may vary with different levels of implementation.

#### Long term costs

We will estimate the longer term costs associated with the trial population using the UKPDS model developed by the University of Oxford [26]. Data on the relevant parameters will be collected during the trial and their values used to model and project longer term costs.

## Discussion

The problems with external validity of trials, and in particular, the problem of highly selected trial populations have been highlighted recently [27]. In this pragmatic trial we have kept the exclusion criteria to a minimum with the goal of maximising external validity and ensuring the results generalise to the target population. With this in mind we have also endeavoured to minimise the response burden on participants while capturing outcomes of interest to patients and their clinicians. The issue of additional work as a result of participating in a trial is also extremely pertinent to the staff in participating general practices. English general practice is under enormous strain [28], and if a practice is struggling to deliver core services, there may be significant capacity issues limiting staff ability to adhere to trial protocols.

The results of this trial will add to the evidence base on different models of supporting self-management of patients with T2DM, and that on the effectiveness of web-based programmes for long term conditions. The results are likely to be of interest to policy makers, clinicians and commissioners, particularly in view of the urgent need to increase uptake of self-management education in people with T2DM.

## Abbreviations

BMI: Body Mass Index
BP: Blood Pressure
CACE: Complier Average Causal Effect
CRF: Case Report Form
DESMOND: Diabetes Education and Self-Management for Ongoing and Newly Diagnosed
DMSES: Diabetes Management Self-Efficacy Scale
DTSQ: Diabetes Treatment Satisfaction Questionnaire
EQ-5D: European Quality of Life Five Dimension Scale
GCP: Good Clinical Practice
GP: General Practitioner
HADS: Hospital Anxiety and Depression Scale
HbA1c: Glycated haemoglobin
HeLP-Diabetes: Healthy Living for People with type 2 Diabetes
HDL: High-density lipoprotein
NHS: National Health Service
NICE: National Institute for Health and Care Excellence
NIHR: National Institute of Health Research
NoCLor: North Central London Research Consortium
NPT: Normalization Process Theory
PCRN: Primary Care Research Network
PRIMENT CTU: Primary Care and Mental Health Clinical Trials Unit
PSSRU: Personal Social Services Research Unit
QALY: Quality-Adjusted Life Year
RCT: Randomised Controlled Trial
SDs: Standard Deviations
SMP: Self-Management Programme
SOPs: Standard Operating Procedures
T2DM: Type 2 Diabetes Mellitus
TSC: Trial Steering Committee
URL: Uniform Resource Locator

## References

[1] IDF Diabetes Atlas: 2014 update [http://www.idf.org/diabetesatlas].

[2] Diabetes UK. Diabetes: Facts and Stats. Version 3. 2014. https://www.diabetes.org.uk.

[3] National Collaborating Centre for Chronic Conditions. Type 2 diabetes: national clinical guideline for management in primary and secondary care (update). Royal College of Physicians; 2008. pp. 1 – 279. 21678628

[4] Hex N, Bartlett C, Wright D, Taylor M, Varley D (2012). Estimating the current and future costs of Type 1 and Type 2 diabetes in the UK, including direct health costs and indirect societal and productivity costs. DiabetMed.

[5] Ellis SE, Speroff T, Dittus RS, Brown A, Pichert JW, Elasy TA (2004). Diabetes patient education: a meta-analysis and meta-regression. Patient EducCouns.

[6] Deakin T, McShane CE, Cade JE, Williams RD (2005). Group based training for self-management strategies in people with type 2 diabetes mellitus. Cochrane Database SystRev.

[7] Davies MJ, Heller S, Skinner TC, Campbell MJ, Carey ME, Cradock S (2008). Effectiveness of the diabetes education and self management for ongoing and newly diagnosed (DESMOND) programme for people with newly diagnosed type 2 diabetes: cluster randomised controlled trial. BMJ.

[8] Deakin TA, Cade JE, Williams R, Greenwood DC (2006). Structured patient education: the diabetes X-PERT Programme makes a difference. DiabetMed.

[9] Newbronner L, Chamberlain R, Borthwick R, Baxter M, Sanderson D (2013). Sustaining and spreading self-management support: lessons from Co-creating Health phase 2.

[10] Khunti K, Gray LJ, Skinner T, Carey ME, Realf K, Dallosso H (2012). Effectiveness of a diabetes education and self management programme (DESMOND) for people with newly diagnosed type 2 diabetes mellitus: three year follow-up of a cluster randomised controlled trial in primary care. BMJ.

[11] Office of National Statistics. Internet Access – Households and Individuals 2014. In. London; 2014: 1–50. http://www.ons.gov.uk/ons/rel/rdit2/internet-acess---households-and-individuals/2014/stb-ia-2014.html.

[12] Pal K, Eastwood SV, Michie S, Farmer AJ, Barnard ML, Peacock R (2013). Computer-based diabetes self-management interventions for adults with type 2 diabetes mellitus. CochraneDatabaseSystRev.

[13] Corbin JM, Strauss A (1988). Unending Work and Care.

[14] May CR, Mair F, Finch T, MacFarlane A, Dowrick C, Treweek S (2009). Development of a theory of implementation and integration: Normalization Process Theory. ImplementSci.

[15] Welch G, Weinger K, Anderson B, Polonsky WH (2003). Responsiveness of the problem areas in diabetes (PAID) questionnaire. DiabetMed.

[16] Eigenmann CA, Colagiuri R, Skinner TC, Trevena L (2009). Are current psychometric tools suitable for measuring outcomes of diabetes education?. DiabetMed.

[17] Zigmond AS, Snaith RP (1983). The hospital anxiety and depression scale. Acta PsychiatrScand.

[18] Bijl JV, Poelgeest-Eeltink AV, Shortridge-Baggett L (1999). The psychometric properties of the diabetes management self-efficacy scale for patients with type 2 diabetes mellitus. JAdvNurs.

[19] Bradley C, Lewis KS (1990). Measures of psychological well-being and treatment satisfaction developed from the responses of people with tablet-treated diabetes. DiabetMed.

[20] Rabin R, de Charro F (2001). EQ-5D: a measure of health status from the EuroQol Group. AnnMed.

[21] Kinmonth AL, Woodcock A, Griffin S, Spiegal N, Campbell MJ (1998). Randomised controlled trial of patient centred care of diabetes in general practice: impact on current wellbeing and future disease risk. The Diabetes Care From Diagnosis Research Team. BMJ.

[22] White IR, Kalaitzaki E, Thompson SG (2011). Allowing for missing outcome data and incomplete uptake of randomised interventions, with application to an Internet-based alcohol trial. Stat Med.

[23] Becque T, White IR (2008). Regaining power lost by non-compliance via full probability modelling. StatMed.

[24] National Institute for H, Clinical E (2006). Methods for development of NICE public health guidance.

[25] National Institute for H, Clinical E (2008). Guide to the Methods of Technology Appraisal.

[26] Clarke PM, Gray AM, Briggs A, Farmer AJ, Fenn P, Stevens RJ (2004). A model to estimate the lifetime health outcomes of patients with type 2 diabetes: the United Kingdom Prospective Diabetes Study (UKPDS) Outcomes Model (UKPDS no. 68). Diabetologia.

[27] Kennedy-Martin T, Curtis S, Faries D, Robinson S, Johnston J (2015). A literature review on the representativeness of randomized controlled trial samples and implications for the external validity of trial results. Trials.

[28] Royal College of General Practitioners: Patient safety implications of general practice workload. In. London; 2015: 1–11.

